# Progress Note 2024: Curing HIV; Not in My Lifetime or Just Around the Corner?

**DOI:** 10.20411/pai.v8i2.665

**Published:** 2024-03-01

**Authors:** Justin Harper, Michael R. Betts, Mathias Lichterfeld, Michaela Müller-Trutwin, David Margolis, Katharine J. Bar, Jonathan Z. Li, Joseph M. McCune, Sharon R. Lewin, Deanna Kulpa, Santiago Ávila-Ríos, Dázon Dixon Diallo, Michael M. Lederman, Mirko Paiardini

**Affiliations:** 1 Division of Microbiology and Immunology, Emory National Primate Research Center, Emory University, Atlanta, Georgia; 2 Department of Microbiology, Perelman School of Medicine, University of Pennsylvania, Philadelphia, Pennsylvania; 3 Center for AIDS Research, University of Pennsylvania, Philadelphia, Pennsylvania; 4 Ragon Institute of MGH, MIT and Harvard, Cambridge, Massachusetts; 5 Infectious Disease Division, Brigham and Women's Hospital, Boston, Massachusetts; 6 HIV Inflammation and Persistence Unit, Institut Pasteur, Université Paris-Cité, Paris, France; 7 Division of Infectious Diseases, Center for AIDS Research, University of North Carolina at Chapel Hill, School of Medicine, Chapel Hill, North Carolina; 8 Department of Medicine, Perelman School of Medicine, University of Pennsylvania, Philadelphia, Pennsylvania; 9 Brigham and Women's Hospital, Harvard Medical School, Boston, Massachusetts; 10 HIV Frontiers, Global Health Accelerator, Bill & Melinda Gates Foundation; 11 Department of Infectious Diseases, The University of Melbourne at the Peter Doherty Institute for Infection and Immunity, Melbourne, Australia; 12 Victorian Infectious Diseases Service, Royal Melbourne Hospital at the Peter Doherty Institute for Infection and Immunity, Melbourne, Australia; 13 Department of Infectious Diseases, Alfred Hospital and Monash University, Melbourne, Australia; 14 Department of Pathology and Laboratory Medicine, Emory University, Atlanta, Georgia; 15 Centro de Investigación en Enfermedades Infecciosas, Instituto Nacional de Enfermedades Respiratorias, Mexico City, Mexico; 16 SisterLove, Inc., Atlanta, Georgia; 17 Division of Infectious Diseases and HIV Medicine, Case Western Reserve University, Cleveland, Ohio

**Keywords:** HIV, SIV, reservoir, persistence, ART, HIV cure, HIV control

## Abstract

Once a death sentence, HIV is now considered a manageable chronic disease due to the development of antiretroviral therapy (ART) regimens with minimal toxicity and a high barrier for genetic resistance. While highly effective in arresting AIDS progression and rendering the virus untransmissible in people living with HIV (PLWH) with undetectable viremia (U=U) [1, 2], ART alone is incapable of eradicating the “reservoir” of resting, latently infected CD4^+^ T cells from which virus recrudesces upon treatment cessation. As of 2022 estimates, there are 39 million PLWH, of whom 86% are aware of their status and 76% are receiving ART [3]. As of 2017, ART-treated PLWH exhibit near normalized life expectancies without adjustment for socioeconomic differences [4]. Furthermore, there is a global deceleration in the rate of new infections [3] driven by expanded access to pre-exposure prophylaxis (PrEP), HIV testing in vulnerable populations, and by ART treatment [5]. Therefore, despite outstanding issues pertaining to cost and access in developing countries, there is strong enthusiasm that aggressive testing, treatment, and effective viral suppression may be able to halt the ongoing HIV epidemic (ie, UNAIDS' 95-95-95 targets) [6–8]; especially as evidenced by recent encouraging observations in Sydney [9].

Despite these promising efforts to limit further viral transmission, for PLWH, a “cure” remains elusive; whether it be to completely eradicate the viral reservoir (ie, cure) or to induce long-term viral remission in the absence of ART (ie, control; Figure 1). In a previous salon hosted by *Pathogens and Immunity* in 2016 [10], some researchers were optimistic that a cure was a feasible, scalable goal, albeit with no clear consensus on the best route. So, how are these cure strategies panning out? In this commentary, 8 years later, we will provide a brief overview on recent advances and failures towards identifying determinants of viral persistence and developing a scalable cure for HIV. Based on these observations, and as in the earlier salon, we have asked several prominent HIV cure researchers for their perspectives.

**Figure 1.**
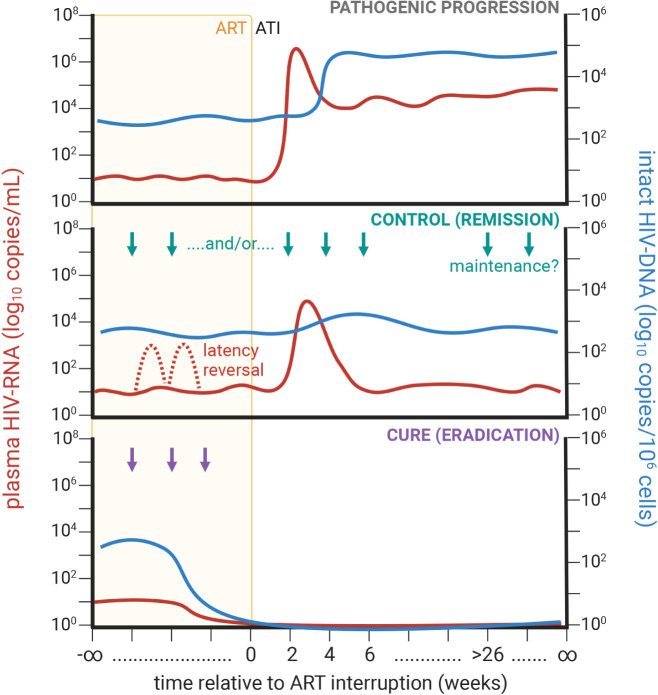
Conceptualization of longitudinal HIV-1 plasma RNA (at left; red line) and tissue DNA content (at right; blue line) during long-term antiretroviral therapy (ART; orange background) and following ART analytical therapy interruption (ATI; white background) for pathogenic disease progression (top; grey) and therapies conferring viral control (middle; green), including potentially with latency reversal (dashed line), or cure (bottom; purple).

## BARRIERS TO A CURE

### Reservoir Establishment

While antiretroviral therapy (ART) suppresses plasma viremia to clinically undetectable levels and blocks *de novo* cellular infection, the reservoir of latently infected cells is remarkably stable during ART [11], thereby facilitating the rapid rebound of systemic viremia upon analytical treatment interruption (ATI) [12, 13]. The latent reservoir is largely formed by the infection of CD4^+^ T cells transitioning from an effector to resting memory state [14] and is established very early on during acute infection as independent events [15–17] with sequences of replication-competent virus during long-term ART reflecting those detected at ART initiation [18]. Early ART initiation is beneficial in limiting viral transmission [19], preserving immune function [20], reducing the size of the viral reservoir [21], and lessening inflammation [22], and as an experimental model to test interventions aimed at disrupting the establishment of viral latency [23]. Outside of effective use as post-exposure prophylaxis (PEP) [16, 24], the mass implementation of very early ART as a cure strategy is doubtful, as the reservoir is established prior to the detection of systemic viral RNA [15, 25, 26] (ie, Fiebig stage I) [27].

### Determinants of Persistence

After undergoing an initial contraction with ART, the viral reservoir expands [28] via clonal expansion by homeostatic [29], antigen-induced [30–32], and possibly integration site-linked proliferation [33–35], which is supported by the survival of quiescent, long-lived cellular viral reservoirs, such as stem cell memory (T_SCM_) CD4^+^ T cells [36, 37]. Chromosomal integration site analyses have demonstrated selection against intact provirus with transcriptional activity during long-term ART [38, 39], which, while occasionally violated by large clones [40], is indicative of host immune surveillance. Moreover, these transcriptionally active viral reservoirs [41, 42], while incapable of *de novo* reseeding amid effective ART, promote the maintenance of HIV-specific CD4^+^ and CD8^+^ T cells [43, 44] and induce a state of functional anergy and impaired differentiation (ie, exhaustion) due to chronic antigenic stimulation. As these latent reservoirs persist for life, much work has been done to comparatively identify determinants of viral control and immune homeostasis in elite controllers (ie, people living with HIV (PLWH) who maintain undetectable viremia in the absence of ART), post-treatment controllers (PTCs; ie, PLWH who control viremia following ATI), and nonhuman primate (NHP) natural hosts (ie, NHPs that do not progress to AIDS despite high levels of viral replication). While non exhaustive (Table 1), many key determinants of viral control and persistence have been identified encompassing an array of mechanisms. It remains to be determined which of these proposed mechanisms is the most essential to facilitate viral control or if they can be simultaneously targeted without toxicity.

**Table 1.** 

Determinant or Correlates of Viral Control^a^	Potential Targets^b^	Cohort^c^	Species^d^
robust, but transient, type-I interferon response [45]	IFNα	natural host	AGM
low inducible CCR5 expression on CD4^+^ T_CM_ [46]	CCR5	natural host	SM
migration of NK cells into the lymphoid B cell follicle [47]	CXCR5	natural host	AGM
blunted LPS responsiveness and reduced cell adhesion [48]	TLR4, ICAM-2	natural host	SM
increased NK cell terminal differentiation [49]	NKG2a	natural host	AGM
homing of CD8 T cells to lymphoid B cell follicle [50]	CXCR5	EC	RM
reduced dysbiosis of the gut microbiota [51]	microbiome	EC	Human
metabolic plasticity of HIV-specific CD8^+^ T_CM_ [52]	IL-15	EC	Human
differentiation, polyfunctionality of HIV-specific CD8^+^ T cells [53]	TOX, TCF-1	EC	Human
viral integration in heterochromatin regions [54]		EC	Human
immune selection on intact proviruses [55]	Nef	EC	Human
stemness, survival, and plasticity of HIV-specific CD8^+^ T cells [56]	WNT, TCF-1, mTORC	EC	Human
low reservoir content in CD4^+^ T_N_ and T_CM_ [57]	early ART	PTC	Human
low reservoir content during ART [58]		PTC	Human
low reservoir content and gut Th17 cell homeostasis [59]		PTC	RM
Env-specific memory B cell responses [60]		PTC	Human
low reservoir content, dominated by CD4^+^ T_TM_ and T_EM_ [61]	early ART	PTC	Human

Determinant or Correlates of Viral Persistence	Potential Targets	Status	Species
exhaustion of HIV-specific CD4^+^ and CD8^+^ T cells [62, 63]	PD-1, CTLA-4	pathogenic	Human
viral persistence in lymphoid CD4^+^ T_FH_ [64]	PD-1	pathogenic	Human
resistance to cytolytic killing [65]	BCL-2	pathogenic	Human
IL-10 related CD4^+^ T cell survival [66]	IL-10, STAT3	pathogenic	RM
CD8^+^ T cell-mediated non-cytolytic control of latency [67]	WNT, TGF-β, β-catenin	pathogenic	Human

a Proposed mechanism of viral control status: central memory T cell (T_CM_), lipopolysaccharide (LPS), natural killer (NK), naïve T cells (T_N_), T helper 17 (Th17), transitional memory T cells (T_TM_), effector memory T cells (T_EM_), follicular T helper cell (T_FH_).

b Receptors or signaling cascades regulating viral control status, if identified.

c Type of viral control status: elite controller (EC), post-treatment controller (PTC).

d Species of cohort: African green monkeys (AGMs), rhesus macaques (RM).

### Markers of Infected Cells

The cellular tropism of HIV is primarily CD4^+^ T cells, and to a lesser extent macrophages [68], based on their expression of CD4, the primary receptor for viral entry, and the CCR5 and CXCR4 co-receptors, which identify the virus as R5 or X4 tropic, respectively; however, selective biomarkers for identifying infected cells remain elusive [69–72]. The use of microfluidic and single-cell sequencing approaches has enabled the isolation of unstimulated blood and lymph node CD4^+^ T cells harboring HIV-DNA, including those with only intact provirus, from PLWH on long-term ART. These analyses reveal that infected cells exhibit transcriptomic signatures favoring HIV silencing, cell survival, and proliferation [73], and clusters of surface receptors associated with immune checkpoint signaling, cell survival, and resistance to cytotoxic killing [74]. Yet, no individual marker, including CCR5, selectively discriminated CD4^+^ T cells harboring HIV-DNA. Likewise, the antibody-mediated depletion of the entire CD4^+^ T cell compartment during ART reduced the absolute numbers, but not the frequency, of infected cells and failed to limit viral recrudescence, with or without reconstitution of CD4^+^ T cells prior to ATI [75, 76]. Therefore, it is likely the only currently feasible route to selectively target infected cells is by their expression of viral envelope (Env) proteins on their cell surface, thereby rendering latently infected cells impervious to detection during ART barring the stimulation of viral replication by latency reversing agents (LRAs).

## PROGRESS TOWARDS A CURE

### Immunotherapy-based Approaches

**Figure 2.**
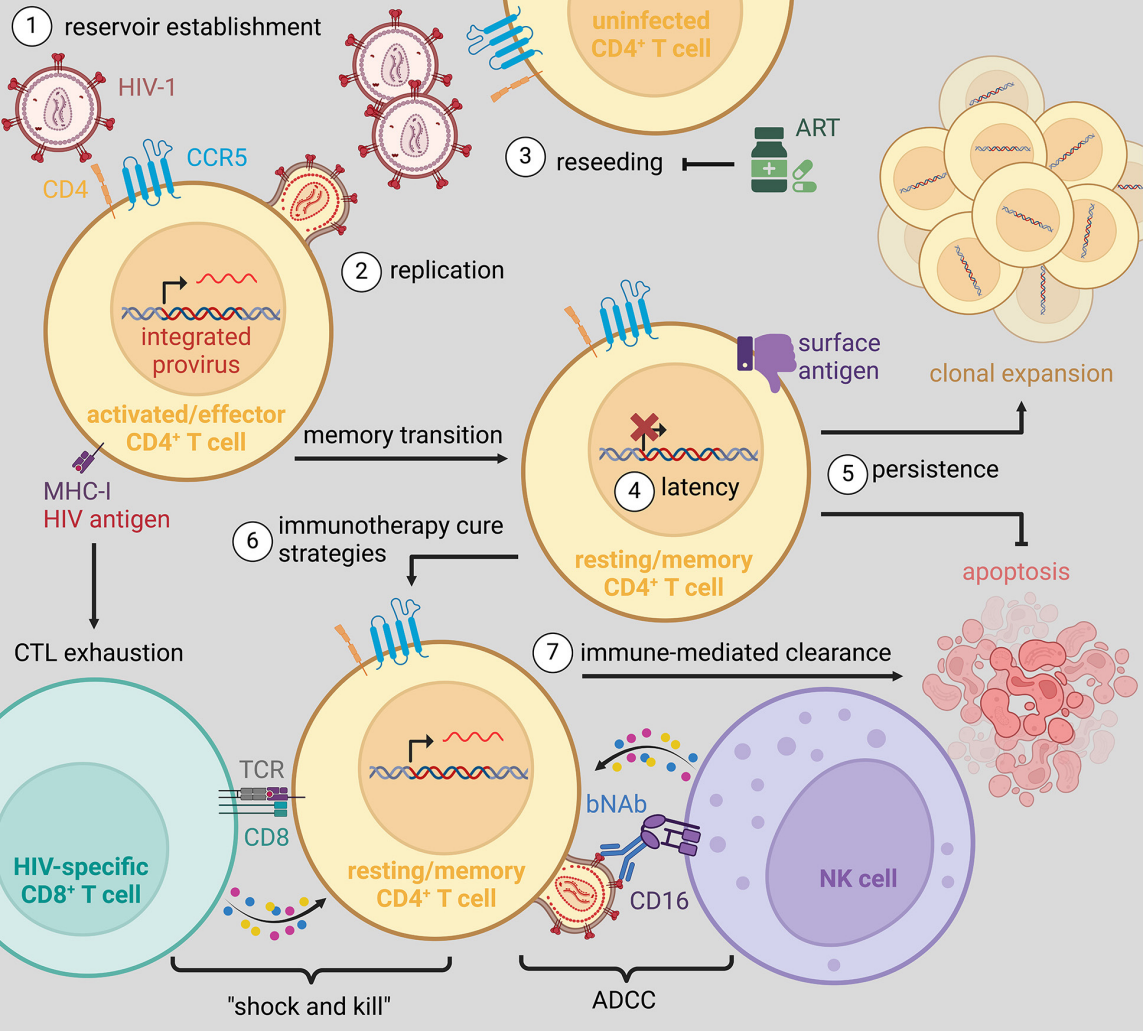
(1) CCR5^+^ CD4^+^ T cells are efficiently infected with HIV-1 resulting in the establishment of a pool of productively infected cells. (2) These highly activated effector CD4^+^ T cells support robust levels of viral replication facilitating the rapid escape from cytolytic T lymphocyte (CTL) responses and inducing a state of CTL exhaustion due to chronic antigenic stimulation. (3) Viral replication leads to the rapid, exponential infection of bystander CD4^+^ T cells and systemic viremia that progressively contributes to CD4^+^ T cell depletion and AIDS progression. Alternatively, antiretroviral therapy (ART) is highly effective in blocking *de novo* infection of vulnerable cells, thereby indirectly reducing plasma viremia as productively infected cells turn over due to viral cytopathic effects and/or immune-mediated clearance. (4) A subset of productively infected effector CD4^+^ T cell reverts to long-lived, resting, memory cells in which viral latency is established. (5) During long-term ART, latently infected memory CD4^+^ T cells persist indefinitely. This persistence has been linked to clonal expansion via homeostatic proliferation, inhibition of apoptotic pathways (ie, prosurvival), and impaired immunosurveillance. (6) Some immunotherapy cure strategies seek to transiently reverse viral latency to enhance viral peptide presentation and the expression of viral envelope on the cell surface (ie, “shock”), thus rendering latently infected cells susceptible to elimination by HIV-specific CD8^+^ T cells or by natural killer (NK) cell mediated antibody-dependent cellular cytotoxicity (ADCC; ie, “kill”), respectively. (7) To promote the immune-mediated clearance of reactivated cells, in an environment where ART protects uninfected cells from infection, combination therapies may be applied to augment the cytotoxicity, activation, homing, and/or differentiation of responding CD8^+^ T cells and NK cells.

Based on these considerable barriers enabling viral persistence during ART, cure strategies have been developed to reverse viral latency, thereby rendering infected cells susceptible to immune clearance (ie, “shock and kill”; Figure 2) [77]. While numerous immunotherapies have been validated as LRAs *in vivo*, these approaches failed to substantially reduce the size of the viral reservoir and demonstrated very limited efficacy at the maximum tolerable dose in PLWH (eg, histone deacetylase (HDAC) inhibition [78] and IL-15 superagonists [79]). While initially promising in pre-clinical models, many LRAs have thus far failed to progress to clinical trials due to safety concerns (eg, CD8 depletion [80], combination immune checkpoint blockade [81, 82], and the inhibition of noncanonical NF-κB signaling via administration of second mitochondria-derived activator of caspase, SMAC, mimetic [83, 84]) or have shown inconsistent responses (eg, TLR7 agonists) [85–87]. Recently, the administration of recombinant IL-2 (Aldesleukin) to ART-suppressed PLWH (n=9) induced a 26-fold increase in plasma viremia 3 days after a 4-day cycle (NCT03308786); however, toxicities resulted in interruption of the trial. Insofar as some latently infected cells exhibit a high barrier to expression [88, 89], it is unlikely currently utilized LRAs will be capable of inducing viral production from all replication-competent proviruses, a major limitation of the “shock and kill” approach, as a rare replication-competent virion can initiate systemic viral recrudescence [90]. Consistent with this constraint, LRA-mediated reductions in viral burden during ART are insufficient to affect viral rebound kinetics upon treatment interruption [81]. Therefore, in the absence of durable immune-mediated viral control, it is unlikely the “shock and kill” approach alone is a viable pathway towards an eradicative cure. Alternatively, given the low inducibility of some integrated viruses, an opposing approach under investigation is to induce potent epigenetic silencing to prevent viral recrudescence in the absence of ART (“block and lock”) [91].

Other immunotherapy-based strategies currently under investigation include disrupting the establishment of viral latency in acute infection and/or at ART initiation and intercepting viral recrudescence following ATI to establish durable immune control. For example, PD-1 blockade in combination with IL-10 neutralization at ATI suppresses viral replication post rebound, albeit with variable responsiveness and significant toxicity [92]. Although the exact mechanisms are still to be determined, this study provides a proof-of-concept that those pathways can be harnessed to tilt the balance towards immune control of a highly replicative virus, such as SIVmac_239_. Given the absence of effective vaccines [93–96] or therapies to elicit durable and potent HIV-specific T cell responses able to control the rebounding virus, next-generation broadly neutralizing antibodies (bNAbs) directed against the viral envelope have emerged as a leading candidate to eliminate cells supporting viral replication via natural killer (NK) cell-mediated antibody-dependent cellular cytotoxicity (ADCC; Figure 2). When administered during ART in combination with LRAs, bNAb therapy permits the targeted labeling and clearance of infected cells supporting viral reactivation [84]; however, the same limitations regarding incomplete induction outlined with “shock and kill” strategies still apply. When given at ATI or during chronic infection, bNAbs are efficacious in suppressing, but not preventing, viral rebound upon either treatment cessation or viral escape amid ongoing therapy [97–103]. While combination bNAb regimens with broad epitope coverage have proven effective in limiting viral escape [104–108], there is considerable interest in identifying if there is complementarity between bNAb-targeted epitopes, such that escape from one bNAb enhances sensitivity to others (eg, inverse resistance pathways), a phenomenon previously observed with specific ART regimens [109]. It has been suggested that bNAbs may also exert a vaccinal effect to support HIV-specific T cell immunity [110, 111], although this is a matter of extensive debate in the field. Irrespective, bNAbs alone are insufficient to purge the pool of latently infected cells, and further studies are needed to assess synergy with other treatment modalities towards controlling HIV in the absence of ART.

### Gene Therapy-based Approaches

**Figure 3.**
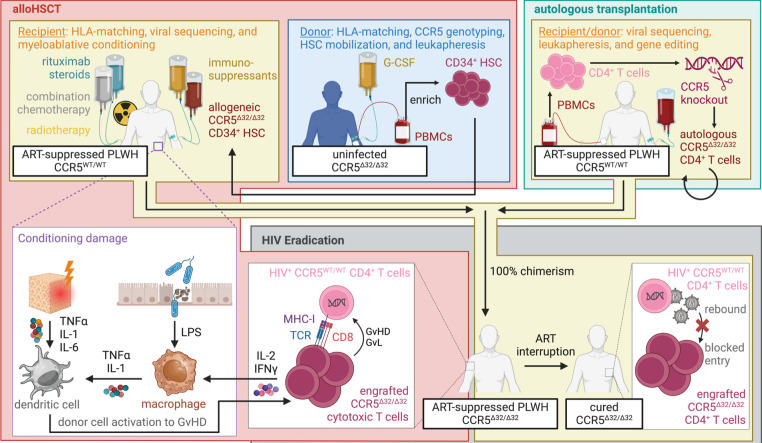
Schematic of the workflow for hematopoietic stem cell transplantation (HSCT) to an ART-suppressed PLWH with wild-type CCR5 (yellow background). Allogeneic HSCT (alloHSCT; red background) is performed using mobilized CD34^+^ HSCs isolated from the blood of an uninfected CCR5^Δ32/Δ32^ donor (blue background) and administered to the patient following conditioning with chemotherapy, radiotherapy, immunosuppressants, and/or immunomodulators to promote cell engraftment and to prevent graft rejection. The alloHSCT conditioning regimen induces systemic tissue damage and a breakdown of the gut epithelium resulting in the production of pro-inflammatory cytokines (IL-1, IL-6, and TNFα) and the influx of bacterial elements, such as lipopolysaccharides (LPS), causing robust dendritic cell and macrophage activation. Autologous transplantation (green background) is performed using CD4^+^ T cells isolated from the blood of the ART-suppressed PLWH. Cells for autologous transplantation then undergo *ex vivo* gene editing to knock out CCR5 and are then re-infused to the donor. Eradication of the HIV reservoir (grey background) occurs upon complete chimerism with donor cells. Infected CD4^+^ T cells are subject to clearance by activated donor cytotoxic T cells against minor recipient antigens as a graft-versus-host disease (GvHD) response. Upon ART interruption, engrafted donor CCR5^Δ32/Δ32^ CD4^+^ T cells are refractory to infection if virus is produced by a residual reservoir.

Despite the complexity of the mechanisms related to viral persistence and the shortcomings with immunotherapy-based interventions, there is evidence that a cure is theoretically possible. For example, long-term viral control following ATI has been observed in 5 patients who underwent allogeneic hematopoietic stem cell transplantation (alloHSCT) using cells from donors homozygous for the CCR5Δ32 mutation (CCR5^Δ32/Δ32^) [112] to prevent subsequent viral infection of engrafted CD4^+^ T cells upon attaining complete chimerism (Figure 3): ie, the Berlin [113, 114], London [115, 116], New York [117], Düsseldorf [118], and City of Hope [119] patients. These patients have been defined as cured, and their experience serves as a proof-of-concept that cure is possible. As these are case studies, efficacy is likely highly skewed due to publication bias for positive results, as there are limited reports of patient deaths [120–123] as would be anticipated with alloHSCT. It is also plausible that, in a subset of patients, the CCR5^Δ32/Δ32^ mutation could select for X4 tropic virus resulting in viral escape [124] as observed with CCR5 inhibitors in the absence of suppressive ART [125]. As currently implemented, this strategy is only advisable for management of aggressive leukemias and lymphomas as it requires pre-conditioning regimens with significant toxicity, including total body irradiation, chemotherapy, and immunomodulatory therapies, and entails a substantial risk of graftversus-host disease (GvHD). It should be noted that myeloablative conditioning in and of itself is insufficient to promote viral control [126–128]. Additionally, alloHSCT is poorly scalable as it requires identification of HLA-matched donors possessing the rare CCR5^Δ32/Δ32^ mutation [129, 130].

### But is it Scalable?

To overcome these limitations, gene editing platforms are being adapted to develop “off-the-shelf” products to disrupt CCR5. For example, *in situ* CRISPR editing to disrupt CCR5 [131] or proviral sequences [132] with non-integrating viral delivery vectors have shown promise in facilitating viral reduction in preclinical animal models. The *ex vivo* disruption of CCR5 in blood CD4^+^ T cells using zinc-finger nucleases (ZFNs) [133] accords significant resistance to HIV *in vitro* [134]; yet, autologous infusions of these cells to ART-treated PLWH results in a limited impact on the kinetics of viral rebound with ATI or the size of the viral reservoir following ART re-initiation [135, 136]. Poor efficacy is tentatively linked to the extent of cell engraftment, which may be ameliorated by differentiating cells towards a stem cell memory phenotype prior to infusion [137] or by additional selection mechanisms to enhance host CD4^+^ T cell depletion, such as short periods of ART interruption. A competing hypothesis is that viral elimination in these patients is dependent on allogeneic immune responses, as all but one of the cured CCR5^Δ32/Δ32^ alloHSCT patients exhibited GvHD (Figure 3) [117]. In this regard, limited viral control has been observed in patients with GvHD undergoing alloHSCT with donor cells that are heterozygous (CCR5^Δ32/WT^; Boston, rebounded at 84 and 225 days [90, 138]) or wildtype (CCR5^WT/WT^) for the CCR5 alleles (Minnesota, rebounded at 288 days [139]; Geneva, undetectable as of 20-months of follow-up [140]). In SIV-infected macaques, the formation of GvHD following CCR5^WT/WT^ alloHSCT was proven to be essential in promoting the stepwise clearance of the viral reservoir across tissue compartments resulting in long-term viral remission [141], analogous to graft-versus-leukemia (GvL) elimination of malignant cells [142, 143]. While providing compelling support for the role of allogeneic immunity, these data suggest that GvHD may be a necessary feature for those cure strategies and that alternative strategies trying to avoid GvHD, such as via gene-edited autologous CD4^+^ T cells, may have a more limited impact on eliminating the reservoir. This raises significant concerns given GvHD's complicated morbidity, clinical management [144], and mortality rate, that may further limit scalability.

### Effective Alternatives to a Cure

Stunning progress has been made in the development of antiretrovirals with reduced toxicity and/or high barriers for genetic resistance. This progress includes the development of non-boosted integrase inhibitors (dolutegravir, DTG [145–147]), reduced dosage prodrugs (tenofovir alafenamide, TAF [148, 149]), nucleoside reverse transcriptase translocation inhibitors (NRTTIs; islatravir [150–152]), capsid assembly inhibitors targeting multiple steps of viral replication (lenacapavir [153, 154]), and nonnucleoside reverse transcriptase inhibitors (NNRTIs) that induce cytotoxicity in infected cells (ie, targeted activator kill, TACK [155]), which have expanded the armamentarium of effective antiretroviral strategies. While inconsistent ART adherence leading to resistance remains problematic, advances in the development of investigational long-acting, slow-effective release (LASER) ART may enable many ART-suppressed PLWH to transition from daily oral regimens to FDA-approved injectable regimens administered monthly (cabotegravir plus rilpivirine) [156–158] or regimens including twice yearly injections (lenacapavir) [154, 159, 160].

If ART suppression of virus replication permits a stable disease course without comorbidities and blocks viral transmission, can this be considered as remission? Or if a cure is required to reduce stigmatization, how does one balance this need against the potential for therapy-related reductions in quality of life, particularly if long-term maintenance therapy is required? Likewise, assuming a prospective cure does not prevent subsequent re-infection, should all persons who experience a cure be maintained on antiretroviral agents? The advances in treatment of HIV as a chronic disease should substantially raise the bar by which the effectiveness and suitability of cure strategies are judged.

### The Challenges for and Importance of an HIV Cure

Although we have not yet identified a scalable strategy to control or cure HIV, significant progress has been made in that direction. Thus far, only CCR5^Δ32/Δ32^ alloHSCT has resulted in a reproducible cure [113–119], but this strategy is limited by the availability of CCR5^Δ32/Δ32^ HLA-matched donors and significant morbidity and mortality of the allogeneic transplant recipient [120–123]. The transplantation of autologous gene-edited CD4^+^ T cells has, to date, demonstrated limited efficacy [135, 136], but might be more effective if a selection process (perhaps even periods of ART cessation) promotes their expansion. Other promising approaches will likely use bNAbs in combination with immune modulators to enhance antiviral immunity to selectively intercept and eliminate cells supporting viral replication upon ART cessation. Other compelling combination approaches may be feasible but would require further study and may be complicated by safety concerns: ie, CCR5^WT/WT^ alloHSCT with a CCR5 inhibitor (eg, Maraviroc or Leronlimab) [141] or CCR5^Δ32/Δ32^ CD4^+^ chimeric antigen receptor (CAR) T cells directed against multiple bNAb targets [161].

While there is optimism regarding the potential of combination approaches, it is important to acknowledge that a scalable, clinically well-tolerated therapeutic strategy to either cure or control HIV in the absence of ART is unlikely to occur in the same timescale as has been proposed to end the HIV epidemic (ie, by 2030) [6, 8]. As such, an emerging area of concern is to address how age-related perturbations in immune function and HIV-associated immunosenescence [162–164] intersect with the maintenance of infected cells during long-term ART [165], with the risk of co-morbidities [166-168], and with responsiveness to immune-based cure strategies [169–171]. Moreover, a portfolio of experimental and FDA-approved drugs are under investigation for their ability to either eliminate (ie, senolytics) or alter the function (ie, senomorphic) of senescent cells, including Bcl-2 inhibitors (Venetoclax), JAK1/JAK2 inhibitors (ruxolitinib), mTOR inhibitors (rapamycin), and tyrosine kinase inhibitors (dasatinib) among others [172, 173]; however, further study is required to evaluate these agents for their utility in attenuating or reversing HIV-induced immunosenescence and affecting HIV persistence.

As advances in ART have rendered HIV a manageable chronic disease, we may need to reconsider what is acceptable in pursuit of a cure. ART-suppressed PLWH remain at a small, but demonstrably greater, risk of comorbidities [174], but it is unclear, and a key question the field will need to address, if a cure will diminish that risk or if immune responses are “scarred” due to persistent epigenetic remodeling [175, 176] or other irreversible damage. Additionally, as cure strategies are considered and developed, effects on quality of life and life expectancy must be monitored. Indeed, while morbidities of FDA-approved immunotherapies [177, 178] and myeloablative conditioning [179] are acceptable in life-threatening malignancies, that will not be the case for most PLWH. Moreover, immunotherapy-based approaches may entail long-term maintenance therapy resulting in substantial “financial toxicity” [180–182] relative to modern ART regimens. Like-wise, given their high projected out-of-pocket cost [183] and requirements for significant clinical infrastructure for implementation and adverse event management, it is doubtful that many cure strategies could be successfully implemented in resource-poor settings and may not be a realistic option for all, at least in the immediate future.

To circumvent these limitations, gene editing approaches using CRISPR-Cas9 and genome-integrating viral vectors are being explored to induce long-term endogenous gene expression, a strategy that has been successfully employed to treat sickle cell disease (eg, FDA-approved Casgevy and Lyfgenia) [184, 185]. For example, gene transfer using adeno-associated virus (AAV) vectors has been explored to induce long-term production of bNAbs; yet, while initial results in the NHP model were encouraging (eg, the Miami monkey [186]), efficacy in humans is limited by the generation of anti-drug antibodies (ADAs) and baseline resistance [187, 188]. Likewise, sustained viral control is rare following the passive transfer of bNAbs in PLWH [106, 107]. A less durable alternative, the delivery of mRNA [189, 190] via lipid nanoparticles (LNPs), has been demonstrated as a safe, effective, and economical therapeutic option to temporarily induce transgene expression, as evidenced by the success of the SARS-CoV-2 mRNA vaccines [191, 192]. When combined with emergent modification strategies to target LNPs to specific organs [193, 194], this may represent a compelling approach through which to transiently modulate innate, adaptive, or humoral immune responses.

It is important to recognize that the timeline for developing an effective, simple, and inexpensive cure strategy is unknown and that this strategy may initially result in a net benefit for some, but not all PLWH. Despite these formidable challenges in finding a scalable curative approach for most PLWH, there remains broad interest and need in the community and among HIV researchers for exploring novel therapeutic strategies to cure HIV. Furthermore, as proven for the past 40 years, research in HIV will continue to benefit many fields in innovating science, including the development of cutting-edge diagnostic technologies and treatment modalities for chronic diseases. In the meantime, as many novel strategies will target host elements, PLWH considering engaging in these trials will need to have a realistic understanding of the risks of these approaches as they pursue a cure. We provide below the perspectives of several leading researchers in the field regarding the status and future of HIV cure research.

## COMMENTS BY LEADERS

### Michael R. Betts

The past several years of HIV cure research have seen remarkable novel immunotherapeutic strategies and unfortunate clinical trial setbacks that together complicate the potential for the development of a truly scalable HIV-eradicative cure. Our aspirational goal continues to be a “one pill” therapeutic HIV cure strategy like that available for hepatitis C virus (HCV). However, there does not yet appear to be a simple virological approach to eliminating the entirety of the latent HIV reservoir, at least until gene editing technologies can be developed capable of finding and eliminating the integrated virus from every infected cell in the body. As such, we must consider the value of a stopgap immunological-based functional cure versus the end goal of pharmacological eradicative cure. Like other chronic human viral infections, most notably the herpesvirus family, HIV co-opts host-preserving immunological mechanisms inherently present at the cellular and anatomical level to perpetuate the viral reservoir and prevent its immunological clearance.

Simply reinvigorating an already failed immune response through T cell restimulation, NK cell modulation, bNAb administration, or exogenous cell therapy is unlikely to overcome these issues. The immune system cannot identify and eliminate HIV-infected cells if (1) the virus remains latent, (2) the infected cells are resistant to cell death, and (3) immune effectors with appropriate functional properties cannot access infected cells. Emerging technologies, including CRIS-PR-based gene editing and therapeutic RNA-based immunomodulation, hold promise to address these problems, but until we develop immunomodulatory strategies that effectively circumvent biology, even a functional cure for HIV will remain elusive.

### Mathias Lichterfeld

What can the human immune system do against HIV reservoir cells – the cells that persist despite ART and represent the main barrier to a cure? The traditional answer to this question has been “nothing,” because, in theory, these cells are in a resting state that promotes proviral transcriptional silencing and maintains “viral latency,” protecting these cells from immune recognition. I believe that this concept, which has now been pursued for more than 2 decades, is in need of a profound revision: Newer single-cell or single-genome assays suggest that these cells can be surprisingly vulnerable to immune defenses and that signatures and footprints of immune selection in the viral reservoir cell pool are remarkably evident when high-resolution analysis techniques are used. I think that we now have the proper tools to investigate the immune mechanisms that can recognize and target HIV reservoir cells and that doing so will ultimately permit us to find weaknesses and vulnerabilities of viral reservoir cells that can be effectively exploited for clinical cure interventions. Key to the success of these efforts will be listening to and learning from community members, so that HIV cure research is a project “of the people, by the people, and for the people” living with HIV.

### Michaela Müller-Trutwin

The last decade has shown that the mechanisms leading to remission of HIV differ in some aspect from those of natural control of HIV. Research in recent years highlighted a potentially important role for innate immunity in post-treatment control. While the IFN-α response is not sufficient for avoiding viral rebound, the pressure it exerts on the virus might give the host more time to chime in with additional antiviral arms. Cellular components of the innate immune system, in particular NK cells, might act directly on eliminating reservoir cells. This can probably only happen if viral transcription is active, such as during residual viral replication, replication in anatomical reservoirs, and at treatment interruption, when the virus is in the process of rebounding. Interestingly, NK cells have the potential to migrate into anatomical sanctuaries, such as B cell follicles. NK cells also provide the necessary help to neutralizing antibodies for ADCC. Moreover, their capacity to adapt and develop antigen-specific memory might revolutionize immunotherapies harnessing NK cells. Whether this property can be exploited for HIV cure approaches, for instance through vaccinal or immunotherapies, still needs to be further tested. Even if most of the PLWH do not develop highly efficient NK cells for several reasons, off-the-shelf products could be constructed that could substitute. Transfer of allogeneic NK and chimeric antigen receptor (CAR)-NK cells present a lower risk of GVHD or cytokine release syndrome than CAR T cells. CAR-NK cells are not susceptible to infection and are more easily applicable for off-the-shelf usage as they do not require a strict autologous HLA matching. Collectively, the arsenal of tools that is available for developing an effective cure can be enlarged by harnessing innate immunity, increasing the chances of achieving a scalable cure one day.

### David Margolis

In the last 8 years, much has changed, but some challenges remain daunting. The effort to end the epidemic using tools at hand to treat all PLWH and completely interrupt ongoing transmission is critical but will not address the desires of millions of PLWH. However, significant advances in the understanding of persistent infection, technologies to measure and characterize the latent reservoir, and approaches to attack the sources of viral rebound after ATI have been made. A substantial number of demanding clinical studies have been successfully completed, adding to our knowledge without measurable harm to the altruistic participants who have made this work possible.

We must safely and methodically seek to enlarge on these modest initial advances, while also developing new approaches. A broadly scalable cure strategy is obviously the ultimate goal, but in the near-term, we hope to see proof-of-concept studies that demonstrate the ability to substantially deplete persistent infection using serial latency reversal agents combined with engineered immunotherapies. Ongoing efforts to develop new approaches, such as base editing of proviral DNA, and interventions to blunt the entry of provirus into latency during ART initiation, are likely to contribute to a later wave of clinical advances towards ART-free remission. If we are as persistent as the virus, we are certain to make progress.

### Katharine J. Bar

Since publication of the previous salon, the field has made important discoveries in HIV reservoir dynamics that inform HIV cure strategies currently in clinical trials. First, data indicating that the majority of the persistent CD4^+^ T cell reservoir is established at or near ART initiation have fueled current interventions. Next, HIV-specific immune responses appear to drive decreases in the intact proviral reservoir over the first 5 to 10 years of ART suppression. While insufficient, the consistent trend towards early reservoir decrease implies a baseline level of immune clearance that could be enhanced. Finally, there is evidence that following the early reservoir decline, immune exhaustion, reservoir cells resistant to killing, and cellular proliferation outcompete anti-HIV immune responses leading to stagnation of reservoir clearance. While challenging, this body of work identifies plausible targets of current interventions.

With advances in related fields of vaccinology, cancer immunology, and genetic engineering, current trials aim to block reservoir formation at ART initiation, enhance HIV-specific cellular and humoral immunity via vaccination, immunomodulation, or gene therapy, and limit cellular proliferation. Thus, the goal of a safe, effective, and scalable intervention to induce HIV cure or ART-free virus control remains a substantial, long-term challenge, but I believe we can celebrate the field's tangible strides towards understanding the determinants of HIV persistence as they better illuminate the path forward.

### Jonathan Z. Li

At the start of 1928, the world had no idea that Dr. Alexander Fleming was about to discover penicillin and open the antibiotic era. At the beginning of 2007, the world had no idea that Timothy Ray Brown was about to undergo his stem cell transplant and that an HIV cure was possible. The arc of scientific discovery is fundamentally not a linear process, and the timeline for discovering an HIV cure and ART-free HIV control is unpredictable. Having said that, I am optimistic about the future. On the basic science side, new single-cell techniques are giving us unprecedented resolution of the cellular transcriptomic, proteomic, and metabolic pathways that underpin HIV persistence. This fundamental knowledge is critical to the development of new strategies. In addition, there are a number of exciting approaches being tested in clinical trials, including bNAbs, latency-reversing agents, immunomodulators, HIV silencing strategies, and gene modification. I can't wait to see what 2024 (and beyond) will bring for the field and for our patients!

### Joseph M. McCune

While ART has made HIV disease manageable for many, this is not the case for most – especially for those in resource-limited parts of the world where the prevalence of disease is high, adherence is difficult, and the availability of ART is increasingly uncertain. For these individuals, it is important to find a way to maintain durable viral suppression absent guaranteed provision of and/or long-term adherence to ART [195].

This goal appears to be technically within reach. “Single shot” (*in vivo*) interventions targeting the liver are already poised to provide clinical benefit for millions living with chronic conditions such as hemophilia [196] and hyperlipidemia [197]. Emerging in parallel are vectors enabling *in vivo* targeting of long-lived cells that are even more relevant to an HIV “cure,” eg, hematopoietic stem cells, B cells, and T_SCM_ cells. Innovative approaches to edit or add genes using only mRNA are opening the door to insertion of membrane-associated and secreted Env-antagonists into the safe harbor of CCR5, thereby also knocking it out. Better understanding of protective CD8^+^ T cell responses in “elite controllers” who suppress HIV absent ART [198] suggests the design of therapeutic vaccines to induce analogous, suppressive T cell responses in non-controllers. Most likely in combination, low-touch interventions such as these may well provide durable viral suppression in the absence of ART.

If so, it will then be necessary to assure accessibility, affordability, and acceptability. While the COVID pandemic has demonstrated that it is possible to scale non-viral vectors carrying mRNA cargos, it has also highlighted gross iniquities in healthcare distribution around the world. Eradication of HIV disease will require the distribution of “curative interventions” that prevent disease progression, block infection upon re-exposure [199], and curtail transmission to those at risk. Aspirational as this goal may seem to be, now is the time for partnerships to form [200, 201] so that such interventions can be advanced for the benefit of all.

### Sharon R. Lewin

Over the last 8 years, progress in cure research has been substantial. These advances have been significantly accelerated by new technologies, such as single-cell sequencing, and in the future, I anticipate further advances as a result of mRNA and lipid nanoparticle technologies that can be used to deliver gene editing directly *in vivo*. In work led by the International AIDS Society to define a target product profile for an HIV cure, which included widespread consultation with the community, the bar is high. There is an expectation that a cure intervention will have minimal toxicity, high efficacy, and also protect from re-infection. I feel we are far off achieving this currently. However, there is no doubt we have made some advances. Several clinical trials of immunomodulatory interventions, administered at the time of viremia (either at ART initiation or ART interruption), have been shown to induce viral control in roughly one third of participants.

This has been described with combination bNAbs either alone or with another immunomodulatory agent and more recently in a small randomized clinical trial of low-dose anti-PD-1 mAbs. To me, these findings are exciting, as it is the first positive signal showing it is possible to induce viral control in some PLWH. There is much more work to be done to understand the mechanism of control and why only some PLWH can achieve this outcome. I am also excited about the advances in *in vivo* gene therapy using viral vectors, but in the future, we will be using mRNA. The induction of long-term antibody production using insertional gene therapy is feasible and showing promise in some macaque models. No matter how hard or complex the science, a cure is of no value if it cannot be scaled and available globally at a reasonable cost. These features must remain front and center, at the same time as we advance the science.

### Deanna Kulpa

The persistent inflammation and immune activation associated with HIV infection not only can lead to T cell exhaustion and senescence but also can complicate the development of latency reversal treatments that rely on T cell responses for both the induction of viral replication from its latent state and the elimination of these reactivated latently infected cells by either viral cytopathic effect or immune cell-mediated killing. However, as our understanding of HIV persistence has increased, so too has my optimism for an HIV cure. By specifically characterizing alterations in cellular signaling and metabolic functions that lead to the chronic inflammatory environment and immune cell activation, we can identify targets for therapeutic interventions that may ameliorate the environment for HIV to persist. For example, many of the processes we observe in HIV infection, such as aberrant DNA damage and repair, shortened telomeres, and impaired mitochondrial function, are also associated with biological aging. Recognizing the parallels between HIV and other biological and disease processes provides opportunity to adopt therapeutic interventions that have shown efficacy for other pathologies. As the pace of discovery and development of new technology accelerates, so will our progress to a cure.

### Santiago Ávila-Ríos

Recognizing that complete eradication of HIV is a daunting task and may not now be feasible with the currently available knowledge and technical tools, I am, however, optimistic that we can achieve a “functional cure” during our lifetimes. That is, “a state in which the virus can still be detected, but ART may be withdrawn without virus recrudescence.” Although multiple thoughtful approaches are being pursued, I am convinced that only a combination of strategies will be effective and generalizable to achieve a functional cure. A more comprehensive understanding of the immune system, greatly fueled by ongoing research, and new tools for genetic editing and cell engineering have resulted in significant advances and informative attempts to achieve this objective by using a combination of strategies to manipulate immune cells to be both HIV resistant and more HIV responsive, to allow effector cells to reach anatomical sites rich in HIV reservoirs, to revert (or reinforce!) epigenetic mechanisms of HIV latency, and to counteract virus diversity and resistance / escape.

A challenge remains on how scalable a functional cure strategy will be. Considering the successful examples of the Berlin and London patients, in whom HIV remission was achieved through transplantation of allogeneic cells resistant to HIV infection, it may be possible that curing HIV may require highly personalized, technically demanding, and costly strategies, for which scale is a barrier, considering that 39 million people are living with HIV. The countries most affected by the HIV epidemic may not have resources and infrastructure to mass produce genetically modified cells and other biologics required in these strategies. Beyond the huge basic and translational science issues presented by HIV cure research, implementation science and health equity issues need also to be considered.

Finally, a close interaction with different global communities to recognize their views and visions of HIV cure is necessary. Managing expectations is also important, considering the stigma that living with HIV and taking ART still represent in many parts of the world. Importantly, maintaining and even increasing the efforts and investments on HIV cure research is of capital importance. The relevance and possible risk of participating in HIV cure research needs to be clearly stated and communicated, not only in clinical trials assessing the effectiveness of specific cure strategies, but also in basic research, aiming to gain additional knowledge on HIV persistence and immunopathogenesis, which often involves accessing tissue samples by use of invasive procedures.

### Dázon Dixon Diallo

*C*ommunity engagement in basic science is essential to the success of finding an HIV cure. Community engagement in HIV research has evolved and become a core component of ethical, meaningful, and impactful involvement of individuals and organizations that share a common stake in the effort to find solutions to minimize and eventually eradicate HIV. More importantly, for PLWH, the stakes are high for finding a permanent solution that relieves them of the lifelong burden of a costly medical routine, social stigma, discrimination, and other unique challenges associated with HIV. It takes all players working in creative and collaborative ways to find the cure that will finally and permanently end HIV as a global threat to the wellbeing and quality of life for people who are wedged in the margins of privilege, power, and safety. It has been demonstrated that research in HIV has led the way across many fields of research in innovating science, technology, care, treatment, prevention, and advocacy for health equity and rights. To be sure, if we are to sustain a successful biomedical solution, one way or another, all roads to the end of HIV go through communities, especially those most affected by the epidemic.

Effective community engagement can involve: (1) coordination of eradication strategies in the community; (2) capacity-building with community advisors, strategic partners, and researchers to learn from each other and work together as collaborators; (3) communication of information, education, and learning with the researchers, constituents, and stakeholders; and (4) creation of new pathways for shared learning, translational experiences, and partnership activities. Collaborative approaches, and commitment to serving as a bridge from science to society, from molecules to medicine, can be the heart of community organizations' long-term, intentional relationships with a diversity of laboratory, clinical, and community-based researchers who, together, continue to do everything possible to find a workable cure to HIV and an end to the epidemic.
