# Erratum to: “Progress Note 2024: Curing HIV; Not in My Lifetime or Just Around the Corner? *Pathogens and Immunity*. 2024;8(2):115-157. doi: 10.20411/pai.v8i2.665”

**DOI:** 10.20411/pai.v8i2.696

**Published:** 2024-03-12

**Authors:** Justin Harper, Michael R. Betts, Mathias Lichterfeld, Michaela Müller-Trutwin, David Margolis, Katharine J. Bar, Jonathan Z. Li, Joseph M. McCune, Sharon R. Lewin, Deanna Kulpa, Santiago Ávila-Ríos, Dázon Dixon Diallo, Michael M. Lederman, Mirko Paiardini

**Affiliations:** 1 Division of Microbiology and Immunology, Emory National Primate Research Center, Emory University, Atlanta, Georgia; 2 Department of Microbiology, Perelman School of Medicine, University of Pennsylvania, Philadelphia, Pennsylvania; 3 Center for AIDS Research, University of Pennsylvania, Philadelphia, Pennsylvania; 4 Ragon Institute of MGH, MIT and Harvard, Cambridge, Massachusetts; 5 Infectious Disease Division, Brigham and Women's Hospital, Boston, Massachusetts; 6 HIV Inflammation and Persistence Unit, Institut Pasteur, Université Paris-Cité, Paris, France; 7 Division of Infectious Diseases, Center for AIDS Research, University of North Carolina at Chapel Hill, School of Medicine, Chapel Hill, North Carolina; 8 Department of Medicine, Perelman School of Medicine, University of Pennsylvania, Philadelphia, Pennsylvania; 9 Brigham and Women's Hospital, Harvard Medical School, Boston, Massachusetts; 10 HIV Frontiers, Global Health Accelerator, Bill & Melinda Gates Foundation; 11 Department of Infectious Diseases, The University of Melbourne at the Peter Doherty Institute for Infection and Immunity, Melbourne, Australia; 12 Victorian Infectious Diseases Service, Royal Melbourne Hospital at the Peter Doherty Institute for Infection and Immunity, Melbourne, Australia; 13 Department of Infectious Diseases, Alfred Hospital and Monash University, Melbourne, Australia; 14 Department of Pathology and Laboratory Medicine, Emory University, Atlanta, Georgia; 15 Centro de Investigación en Enfermedades Infecciosas, Instituto Nacional de Enfermedades Respiratorias, Mexico City, Mexico; 16 SisterLove, Inc., Atlanta, Georgia; 17 Division of Infectious Diseases and HIV Medicine, Case Western Reserve University, Cleveland, Ohio

## ERRATUM

The comments provided by Santiago Ávila-Ríos were mistakenly omitted. The article has been corrected to add his comments in the “Comments by Leaders” section, and his name has been included in the list of authors.

